# Restricted fluid bolus volume in early septic shock: results of the Fluids in Shock pilot trial

**DOI:** 10.1136/archdischild-2018-314924

**Published:** 2018-08-07

**Authors:** David Philip Inwald, Ruth Canter, Kerry Woolfall, Paul Mouncey, Zohra Zenasni, Caitlin O’Hara, Anjali Carter, Nicola Jones, Mark D Lyttle, Simon Nadel, Mark J Peters, David A Harrison, Kathryn M Rowan

**Affiliations:** 1 Paediatric Intensive Care Unit, St Mary’s Hospital, Imperial College Healthcare London NHS Trust, London, UK; 2 Clinical Trials Unit, Intensive Care National Audit & Research Centre, London, UK; 3 Department of Psychological Sciences, North West Hub for Trials Methodology, University of Liverpool, Liverpool, UK; 4 Parent representative; 5 Faculty of Health and Applied Sciences, University of the West of England, Bristol, UK; 6 Emergency Department, Bristol Royal Hospital for Children, Bristol, UK; 7 Respiratory, Critical Care and Anaesthesia Section, Institute of Child Health, University College London Great Ormond Street, London, UK

**Keywords:** general paediatrics, intensive care, accident & emergency, resuscitation, infectious diseases

## Abstract

**Objective:**

To determine the feasibility of Fluids in Shock, a randomised controlled trial (RCT) of restricted fluid bolus volume (10 mL/kg) versus recommended practice (20 mL/kg).

**Design:**

Nine-month pilot RCT with embedded mixed-method perspectives study.

**Setting:**

13 hospitals in England.

**Patients:**

Children presenting to emergency departments with suspected infection and shock after 20 mL/kg fluid.

**Interventions:**

Patients were randomly allocated (1:1) to further 10 or 20 mL/kg fluid boluses every 15 min for up to 4 hours if still in shock.

**Main outcome measures:**

These were based on progression criteria, including recruitment and retention, protocol adherence, separation, potential trial outcome measures, and parent and staff perspectives.

**Results:**

Seventy-five participants were randomised; two were withdrawn. 23 (59%) of 39 in the 10 mL/kg arm and 25 (74%) of 34 in the 20 mL/kg arm required a single trial bolus before the shock resolved. 79% of boluses were delivered per protocol in the 10 mL/kg arm and 55% in the 20 mL/kg arm. The volume of study bolus fluid after 4 hours was 44% lower in the 10 mL/kg group (mean 14.5 vs 27.5 mL/kg). The Paediatric Index of Mortality-2 score was 2.1 (IQR 1.6–2.7) in the 10 mL/kg group and 2.0 (IQR 1.6–2.5) in the 20 mL/kg group. There were no deaths. Length of hospital stay, paediatric intensive care unit (PICU) admissions and PICU-free days at 30 days did not differ significantly between the groups. In the perspectives study, the trial was generally supported, although some problems with protocol adherence were described.

**Conclusions:**

Participants were not as unwell as expected. A larger trial is not feasible in its current design in the UK.

**Trial registration number:**

ISRCTN15244462.

**What is already known on this topic?**

- Rapid, liberal fluid bolus resuscitation is integral to the management of children presenting to emergency departments with septic shock.
- No trials have compared a more restricted fluid bolus resuscitation strategy with the currently recommended strategy in high-income countries.
- The optimal amount of fluid for resuscitation for children presenting with septic shock in high-income countries is an important unanswered question.

**What this study adds?**

- The Fluids in Shock (FiSh) pilot compared a restricted fluid bolus volume (10 mL/kg) with the current recommendation (20 mL/kg) to determine the feasibility of a large-scale trial.
- A larger FiSh trial is not feasible; participants had a lower severity of illness than expected.
- Further observational work is required to determine the epidemiology of severe childhood infection in the UK in the postvaccine era.

## Background

Rapid, bolus fluid resuscitation is integral to the management of children presenting with septic shock. The 2009 American College of Critical Care Medicine-Pediatric Advanced Life Support (ACCM-PALS) clinical guideline recommended fluid resuscitation with boluses of 20 mL/kg, up to a total of 200 mL/kg in the first hour.^1^ However, this recommendation is now controversial; it was based on retrospective observational studies, some involving small numbers of children,^2–4^ and audit data have shown that the recommendations are often not followed.^5^

In Africa, a multicentre randomised controlled trial (RCT), the Fluid Expansion as Supportive Therapy (FEAST) trial, compared bolus fluid resuscitation of 20 mL/kg with maintenance fluid in over 3000 children with severe infection.^6^ The study reported 35% higher mortality associated with bolus fluid resuscitation. Although conducted in Africa in a low-income setting, the FEAST trial highlighted the lack of evidence for bolus fluid resuscitation for children in middle-income and high-income settings.^7 8^

No trial has compared restricted bolus fluid resuscitation strategy with recommended bolus fluid resuscitation in children with septic shock in high-income countries. In the UK, children presenting to emergency departments (EDs) with severe sepsis and discussed with a paediatric intensive care retrieval service have a reported mortality of up to 17%.^9^ With emerging data suggesting that excessive fluid administration is associated with worse outcomes in paediatric intensive care unit (PICU),^10–14^ the optimal amount of fluid for resuscitation for children presenting with septic shock remains an important unanswered question.

To address this problem, the Fluids in Shock (FiSh) trial was developed, which aimed to evaluate whether a restricted fluid bolus volume (10 mL/kg), compared with currently recommended fluid bolus volume (20 mL/kg), is associated with improved outcomes for children presenting to UK EDs with presumed septic shock. Our initial qualitative feasibility study results have been reported previously.^15^ This paper reports the results of the external pilot trial and embedded parent and staff perspectives study.

## Methods

### Study design

The study design was a pragmatic, open, multicentre pilot RCT. The pilot trial was sponsored by the Imperial College Healthcare NHS Trust and coordinated by the Intensive Care National Audit & Research Centre Clinical Trials Unit (CTU). The ISRCTN trial registration number is 15244462. The protocol is available at https://www.journalslibrary.nihr.ac.uk/programmes/hta/1304105/#/.

### Pilot trial

#### Sites and participants

Sites were set up in a ‘hub and spoke’ model in three regions in England. The ‘hubs’ were four regional hospitals with PICUs (two hospitals covered the same region), three of which also had an integrated ED. The ‘spokes’ were nine district general hospitals with an ED but not a PICU linked to the ‘hub’ PICU by the regional PICU retrieval service. Participating EDs were research-active sites and part of the Paediatric Emergency Research in the United Kingdom & Ireland network.^16^ Extensive training was provided for the site teams, including a site initiation visit from the CTU and local training of clinical staff by their own research staff.

The inclusion criteria were age older than 37 weeks (corrected gestational age) and younger than 16 years; clinical suspicion of infection; and signs of shock—defined as age-adjusted hypotension (less than fifth centile systolic blood pressure (BP) for age or capillary refill time (CRT) greater than or equal to 3 s)—after receipt of 20 mL/kg of bolus fluid. Fifth centile systolic BP was provided to sites in age bands on wallet-sized cards for quick reference (<1 week: <60 mm Hg; 1 week to <1 year: <70 mm Hg; 1 to <2 years: <75 mm Hg; 2 to <5 years: <80 mm Hg; 5 to <12 years: <85 mm Hg; and ≥12 years: <90 mm Hg). The exclusion criteria were prior receipt of more than 20 mL/kg of bolus fluid; conditions in which bolus fluid resuscitation should be curtailed; or full active resuscitation not within the current goals of care.

#### Randomisation and trial intervention

Eligible patients were randomised while in an acute assessment area (eg, paediatric assessment unit or ED) and allocated 1:1 to either 10 mL/kg or 20 mL/kg boluses over a 4-hour resuscitation period, without prior consent. The resuscitation period was divided into 15 min cycles, with one bolus of either 10 mL/kg or 20 mL/kg to be delivered in each cycle. The maximum amount of fluid that could be given per bolus was either 500 mL (for those allocated to 10 mL/kg boluses) or 1000 mL (for those allocated to 20 mL/kg boluses). Fluid type and other interventions were left to the discretion of the treating clinician. At the end of each cycle, if age-adjusted signs of shock persisted, then another bolus of the same size was given within the next 15 min cycle.

In participants whose shock resolved or who showed signs of fluid overload (pulmonary oedema—rales on auscultation or pulmonary oedema fluid in the endotracheal tube—or new or increasing hepatomegaly), delivery of further fluid boluses was withheld. If, within the 4-hour resuscitation period, fluid boluses were indicated, that is, signs of shock were present in the absence of signs of fluid overload, cycles were recommenced with allocated boluses until the end of the 4-hour intervention period. After this period, any further treatment was at the discretion of the treating clinician.

The maximum amount of fluid that could be given within the pilot trial protocol, regardless of allocation, was 120 mL/kg (excluding the 20 mL/kg bolus prerandomisation).

#### Consent

A member of the site research team approached parents/legal representatives as soon as appropriate after randomisation to take consent for use of study data, according to the guidance developed in the FiSh feasibility study^15^ and elsewhere.^17–21^ This is known as research without prior consent (RWPC), a methodology favoured in emergency care trials.^22 23^ Specific FiSh RWPC methodology was developed during the feasibility study^15^ for all possible situations, including early discharge or death of the participant.

#### Outcome measures

The objectives of the pilot trial were to test if the processes worked together, and to inform the design and conduct of the full FiSh trial (should this be the recommendation from the pilot). Outcome measures were driven by the progression criteria to be assessed by the pilot, and included recruitment and retention, protocol adherence and demonstration of separation between the groups, distribution of potential trial outcome measures, and parent and staff perspectives.

These were determined by the proportion of eligible participants recruited, number of participants recruited per site per month, proportion of parents/legal representatives refusing consent, proportion of fluid boluses delivered at the correct volume and time during the intervention period (at least 80% of bolus fluid resuscitation delivered at correct volume and timing ±10%), total volume of fluid received during the intervention period in each treatment group (absolute total volume of fluid administered during the first hour and first 4 hours is lower (by at least 25%) in the 10 mL/kg group), characteristics of potential outcome measures and observed adverse events, and parent and staff perspectives.

Data collection was via a secure, dedicated, electronic database. Sites collected data throughout each patient’s hospital admission on the inclusion criteria, baseline—including the Paediatric Index of Mortality (PIM2r; the recalibrated version of PIM2 score, a PICU severity of illness score giving a population risk of mortality)^24 25^ interventions, physiology, location of care to hospital discharge and survival at day 30. For participants admitted to PICU, daily organ support data were obtained via linkage with the Paediatric Intensive Care Audit Network, the UK national clinical audit for paediatric intensive care.

#### Statistical analysis

The trial was set up as a small pilot RCT without a defined primary outcome, and hence without a usual power calculation to determine sample size. Instead, sample size was determined to be adequate to estimate the parameters to be tested.^26^ Based on available data, it was anticipated that the 12 EDs would recruit approximately one participant per month, that is, 108 participants over 9 months.

All statistical analyses were documented a priori in a Statistical Analysis Plan (available from https://www.icnarc.org/Our-Research/Studies/Fish/Study-Documents). Statistical analyses were based on the intention-to-treat principle. All tests used were two-sided with significance levels set at p<0.05 and with no adjustment for multiplicity. Final analyses were conducted using Stata/SE V.14.0.

### Embedded perspectives study

This was a mixed-method study which aimed to explore parent and staff experiences and views of the pilot trial. The participants were pilot trial site staff and parents of randomised children. The questionnaire and topic guides were developed using previous research^17^ and feasibility study findings.^15^ Quantitative data were collected from the focus groups via a keypad voting system, alongside audio-recorded qualitative discussions. Informed consent was taken. Interviews continued until no new themes were identified, that is, data saturation was reached.^27^ Qualitative data analysis was performed according to the methodology outlined in the feasibility study.^14^ Quantitative data were analysed using simple descriptive statistics. Data synthesis was pragmatic and drew on the constant comparative approach.^28 29^

## Results

### Pilot trial

#### Sites, participants and recruitment

The pilot trial was conducted in 13 hospitals from July 2016 to April 2017, with follow-up completed on 31 May 2017. In total, 297 patients who had received a fluid bolus of any size were screened. Eighty-eight (29.8%) received a fluid bolus of less than 20 mL/kg, while 108 (51.7%) did not meet the defined clinical signs of shock after receiving 20 mL/kg. There were 18 (17.8%) patients who met one or more exclusion criteria.

**Figure 1:**
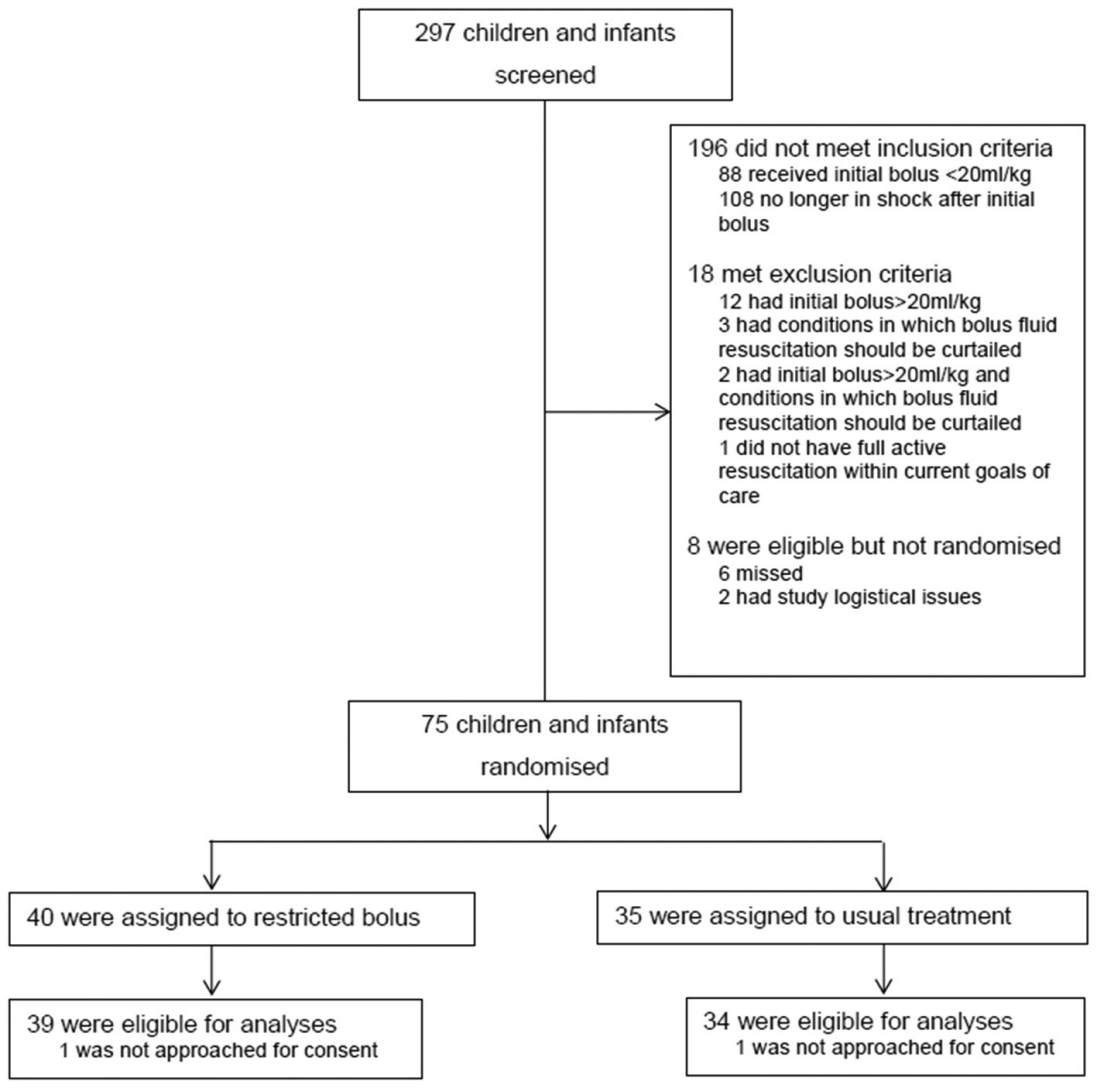
Consolidated Standards of Reporting Trials flow diagram.

Overall, 75 (90.4%) out of 83 eligible patients were recruited, 40 into the 10 mL/kg group and 35 into the 20 mL/kg group. Two were withdrawn (figure 1) as the parents/legal representatives could not be approached for informed consent. The overall recruitment rate was 0.9 participants per site per month (95% CI 0.7 to 1.2), although the majority of recruitment was led by three sites. Recruitment was stopped at the end of the prespecified 9-month period from first site opening.

Baseline characteristics were well matched, although there was some imbalance in age and consequently in weight (table 1). The majority of participants met the shock criteria of CRT≥3 s (76.9% in 10 mL/kg group, 88.2% in 20 mL/kg group). The PIM2r score was balanced across treatment groups but was lower than expected. The mean PIM2r score was 2.1%.

**Table 1:** Baseline characteristics of patients by treatment group

Characteristics	10 mL/kg (n=39)	20 mL/kg (n=34)
Age (months)		
Median (IQR)	11 (1–35)	2 (1–17)
Gender, n (%)		
Male	24 (61.5)	18 (52.9)
Female	15 (38.5)	16 (47.1)
Weight (kg)		
Median (IQR)	9 (5–13)	5 (4–10)
Shock criteria met, n (%)		
CRT* only	30 (76.9)	30 (88.2)
Hypotension only	2 (5.1)	2 (5.9)
CRT* and hypotension	1 (2.6)	2 (5.9)
Neither	6 (15.4)	0 (0.0)
Systolic BP (mm Hg)		
Median (IQR)	102 (91–114)	104 (89–115)
CRT (s)		
Median (IQR)	3 (3–4)	3 (3–4)
PIM2r (2016) score (%)		
Median (IQR)	2.1 (1.6–2.7)	2.0 (1.6–2.5)
Infection confirmed, n (%)		
No	19 (48.7)	17 (50.0)
Yes	20 (51.3)	17 (50.0)
Organism		
Bacterial, n (%)	5 (31.3)	8 (50.0)
*Escherichia coli*	1 (6.3)	4 (25.0)
*Enterococcus faecalis*	2 (12.5)	0 (0.0)
Group A streptococcus	1 (6.3)	1 (6.3)
Group B streptococcus	0 (0.0)	1 (6.3)
*Staphylococcus aureus*	1 (6.3)	0 (0.0)
*Meningococcus*	0 (0.0)	1 (6.3)
Gram-positive coccus (unspecified)	0 (0.0)	1 (6.3)
Viral, n (%)	10 (62.5)	8 (50.0)
Respiratory syncytial virus	5 (31.3)	3 (18.8)
Rhinovirus	2 (12.5)	3 (18.8)
Influenza A	0 (0.0)	1 (6.3)
Metapneumovirus	1 (6.3)	0 (0.0)
Enterovirus	1 (6.3)	1 (6.3)
Rotavirus	1 (6.3)	0 (0.0)
Others, n (%)	1 (6.3)	0 (0.0)
Falciparum malaria	1 (6.3)	0 (0.0)

*CRT≥3 s.

BP, blood pressure; CRT, capillary refill time; PIM2r, Paediatric Index of Mortality, recalibrated version.

Thirty-seven participants (51.3% in 10 mL/kg group, 50% in 20 mL/kg group) had infection confirmed by the site team, that is, positive bacterial, viral or fungal microscopy, culture, PCR or immunofluorescence test. However, in five of these, the organism was not recorded. Of the remaining 32, 13 had bacterial infections in sterile sites (table 1), only one of which was vaccine-preventable. There were 18 viral infections, most of which were respiratory pathogens. One patient had falciparum malaria.

#### Adherence to protocol and separation

All participants randomised to the 20 mL/kg group received their first bolus. Three participants randomised to the 10 mL/kg group did not receive their first bolus, although correctly identified as in shock, because it was deemed they no longer required fluid postrandomisation. Of the subsequent boluses, one participant in the 20 mL/kg group was administered a bolus when the shock criteria were not met. Five participants in total (three from the 10 mL/kg group and two from the 20 mL/kg group) who met the shock criteria did not receive the designated fluid bolus (table 2).

**Table 2:** Protocol deviations by treatment group

Variables		10 mL/kg (n=39)	20 mL/kg (n=34)
Did not receive first bolus	Patients, n (%)	3 (7.7)	0 (0.0)
Of subsequent boluses			
Bolus given, shock criteria not met	Deviations, n	0	1
	Patients, n (%)	0 (0.0)	1 (2.9)
Shock criteria met, no bolus given	Deviations, n	3	2
	Patients, n (%)	3 (7.7)	2 (5.9)

The majority of participants in both groups received one fluid bolus, 23 (59%) in the 10 mL/kg group and 25 (74%) in the 20 mL/kg group. During the total 4-hour intervention period, only four patients received four or more fluid boluses. Seventy per cent of participants required only one study fluid bolus and only 11% required more than two study boluses.

Assessment of the separation progression criteria showed that the mean total volume of study fluid given during the first hour was 38% lower (13.5 vs 20.7 mL/kg) and during the entire 4-hour intervention period was 44% lower (mean 14.5 vs 27.5 mL/kg) in the 10 mL/kg group compared with the 20 mL/kg group. At the end of the 4-hour intervention period, this corresponded to a statistically significant mean difference of −11.2 mL/kg (95% CI –16.6 to –5.8 mL/kg; p<0.001). With regard to the adherence progression criteria, overall, 37 (78.7%) of 47 boluses were delivered at the correct volume and within 15 min in the 10 mL/kg group, whereas 24 (54.5%) of 44 boluses were delivered at the correct volume and within 15 min in the 20 mL/kg group (table 3).

**Table 3:** Treatment delivery by group

Variables	10 mL/kg	20 mL/kg
Patients, n	39	34
Number of study boluses delivered, n (%) of patients		
0	3 (7.7)	0 (0.0)
1	23 (59.0)	25 (73.5)
2	8 (20.5)	6 (17.6)
3	3 (7.7)	1 (2.9)
4 or more	2 (5.1)	2 (5.9)
Total volume of study fluid received during the first hour (mL/kg)*		
Mean (SD)	13.5 (8.0)	20.7 (8.3)
Total volume of study fluid received during the intervention period (mL/kg)*		
Mean (SD)	14.5 (11.1)	25.7 (12.0)*
Total number of boluses delivered†	58	48
Volume of study bolus, n (%) of boluses		
<10 mL/kg‡	2 (3.4)	3 (6.3)
10 mL/kg†	56 (96.6)	5 (10.4)
20 mL/kg†	0 (0.0)	40 (83.3)
Timing of delivery of study bolus, n (%) of boluses§		
≤15 min	38 (80.9)	30 (68.2)
16–20 min	7 (14.9)	5 (11.4)
21–30 min	2 (4.3)	1 (2.3)
>30 min	0 (0.0)	8 (18.2)
Delivery of study bolus with 15 min by age group, n (%) of boluses‡		
<1 year	24/27 (88.9)	22/29 (75.9)
1 to <2 years	7/9 (77.8)	2/3 (66.7)
2 to <5 years	6/7 (85.7)	2/4 (50.0)
≥5 years	1/4 (25.0)	4/8 (50.0)
Study fluid boluses delivered at the correct volume and within 15 min, n (%) of boluses‡		
No	10 (21.3)	20 (45.5)
Yes	37 (78.7)	24 (54.5)

*P<0.001 (t-test).

†All study boluses were of normal saline except 4 of plasmalyte and 4 of Hartmann’s solution.

‡±10%.

§Timing of delivery not reported for 15 boluses (11 in 10 mL/kg group, 4 in 20 mL/kg group).

#### Potential outcome measures for a future trial

There were no deaths and no serious adverse events reported. Two patients in the 20 mL/kg group developed signs of fluid overload after the first study bolus, and consequently did not receive a second bolus despite remaining in shock. Overall, 29% participants were admitted to PICU. As expected in this small pilot trial, length of hospital stay, transfers to PICU, length of stay in PICU and days alive and free of PICU to 30 days did not differ significantly between the groups (table 4).

**Table 4:** Potential outcome measures by treatment group

Potential outcome measures	10 mL/kg (n=39)	20 mL/kg (n=34)	Difference (95% CI)
Hospital mortality, n/N (%)	0/39 (0.0)	0/34 (0.0)	NA
Length of hospital stay (days), median (IQR) (N)	4 (3–7) (39)	5 (4–8) (34)	−1 (−2.5 to 0.5)
Transferred to PICU, n/N (%)	10/39 (25.6)	11/34 (32.4)	−6.7 (−27.6 to 14.1)
Length of stay in PICU (hours), median (IQR) (N)	45 (18–143) (10)	119 (52–228) (11)	−65 (−171 to 41)
Days alive and free of PICU up to 30 days postrandomisation, mean (SD) (N)	28.9 (2.4) (39)	27.9 (3.6) (34)	1.0 (−0.4 to 2.4)
Receipt of mechanical ventilation, n/N* (%)	4/36 (11.1)	8/32 (25.0)	−13.9 (−32.1 to 4.3)
Duration of mechanical ventilation (days), median (IQR) (N)*	6 (4–8) (4)	5.5 (4–8.5) (8)	0 (−5.9 to 5.9)
Days alive and free of mechanical ventilation up to 30 days postrandomisation, mean (SD) (N)*	29.3 (2.1) (36)	28.5 (2.7) (32)	0.8 (−0.4 to 2.0)
Receipt of inotropes, n/N* (%)	1/36 (2.8)	5/32 (15.6)	−12.8 (−26.5 to 0.8)
Mortality at 30 days postrandomisation, n/N (%)	0/39 (0.0)	0/34 (0.0)	NA

*Organ support data missing for three patients in the 10 mL/kg group and two patients in the 20 mL/kg group transferred to PICUs not participating in the pilot trial.

NA, not available; PICU, paediatric intensive care unit.

### Embedded perspectives study

A total of 52 (69%) of 75 parents of randomised participants provided consent to complete a questionnaire or take part in a telephone interview. Of these, 45 (87%) parents (34 mothers, 11 fathers) from 44 families completed a questionnaire before leaving the hospital. Data saturation was reached at 20 (38%) of 52 interviews with parents (19 mothers, 1 father; 26.5 (median) days since child’s admission). There were three pilot trial ‘hub’ site focus groups (20 staff) and telephone interviews with 7 (35%) of 20 invited staff (until data saturation point), including 14 (52%) of 27 nurses and 13 (48%) of 27 doctors from 7 (58%) of 12 sites (for thematic analysis, see online supplementary file).

10.1136/archdischild-2018-314924.supp1 Supplementary file 1

Interview and questionnaire data indicated some parents were surprised to discover that their child had been enrolled into the pilot trial without prior informed consent (online supplementary file). However, clear explanations from site staff about RWPC and the nature of the intervention appeared to elicit parental support.

Site training had prepared staff for recruitment and RWPC. The randomisation method was viewed as straightforward. However, some clinical staff found it difficult to complete the case report form while treating a child. Staff also described problems adhering to the protocol, including administering 20 mL/kg boluses within the 15 min cycles and a lack of equipoise among a minority of clinicians when a child had been randomised to a 20 mL/kg allocation due to concerns about fluid overload.

## Discussion

The pilot trial was successfully conducted. Screening logs showed that over 90% of eligible patients were randomised across the study sites over the study period with a recruitment rate of 0.9 participants per site per month, very close to the anticipated recruitment rate of 1 per site per month.

However, the sites opened over a 4-month period rather than all at once, reducing the anticipated recruitment total from 108 to 84. Recruitment was driven mainly by three study sites, which recruited 38 of the 75 participants. A staggered opening of sites would be needed in the design for a larger FiSh trial, targeting sites most likely to see a high number of eligible patients.

The process of RWPC worked smoothly, with no parents refusing consent. This is likely due to the bespoke FiSh RWPC methodology, developed during the feasibility study.^15^ No participants died during the course of the study, so the procedures to follow in the event of a child’s death were not tested.

The intervention was delivered according to protocol in the majority of participants. Adherence to volume and timing was close to achieving the 80% progression criteria target in the 10 mL/kg group but was not as good in the 20 mL/kg group. This appeared to be due to the difficulty in delivering the 20 mL/kg bolus within the 15 min time frame, especially in the period immediately postrandomisation. There was also some suggestions from the embedded perspectives study, indicating that some clinicians lacked equipoise, favouring 10 mL/kg over 20 mL/kg fluid boluses, despite the recently updated ACCM-PALS guidance continuing to recommend 20 mL/kg boluses.^30^ Despite these challenges, good separation between the groups was achieved, with volume of fluid delivered (in mL/kg) 35% lower in the first hour and 44% lower over the entire 4-hour period in the 10 mL/kg group. If a larger trial were feasible, the resuscitation algorithm could be modified to improve adherence by allowing for longer periods to deliver study boluses.

Though the trial processes were deemed feasible, the population had lower severity of illness than expected from previous data 9, impacting on the feasibility of a larger study. This may be a consequence of the inclusion criteria being too lax in the context of an increasingly immunised population. A recent Europe-wide study demonstrated that the disease burden of severe childhood infection is mainly in children younger than 5 years and is largely due to vaccine-preventable meningococcal and pneumococcal infections.^31^ However, in the UK, the childhood vaccination programme has resulted in massive reductions in the incidence of both group B meningococcal disease^32^ and invasive pneumococcal disease.^33^ Indeed, only one patient recruited into the FiSh pilot trial had a vaccine-preventable infection. Thus, if the FiSh inclusion criteria were to be changed to restrict to more severely ill children, then the number of eligible children would inevitably be reduced, impacting on the likelihood of completing a large-scale trial in an acceptable time frame in the UK.

A recent systematic review^34^ identified only one RCT, other than FEAST, which investigated different-sized fluid bolus therapy in children in septic shock.^35^ However, this was a small, single-centre study in India. Thus, at the time of writing, the optimum strategy for fluid bolus resuscitation in children with septic shock in high-income countries remains unknown.

## Conclusions

A larger FiSh trial, with the current design, and in the UK, is not feasible. Further observational research is required to determine the epidemiology of severe childhood infection in the UK in the postvaccine era.
