# Supplementary material for: Restricted fluid bolus volume in early septic shock: results of the Fluids in Shock pilot trial
Source: Arch Dis Child. 2018 Aug 7;104(5):426–31. doi: 10.1136/archdischild-2018-314924 (PMC6557227; doi:10.1136/archdischild-2018-314924)
Supplement: Supplementary file 1 [file archdischild-2018-314924supp001.pdf]

Additional file

Table. Themes identified in the embedded perspectives study involving parents and site staff

Theme	Subtheme	Qualitative data	Quantitative data
		Example quotes from parent interviews (n=20)	***Parent questionnaire findings (n=45)***
**RWPC in FiSh was acceptable but some concerns**	Initial surprise at RWPC in FiSh	(Discussed in 6 interviews)<br>*I was initially surprised that obviously this had happened, because obviously the consent happened afterwards.* **P12, mother, telephone interview.**<br><br>*Um, and yeah, I think it was a day or two, maybe two days later, um, two people came down and spoke to me about FiSh and I was, um, quite shocked 'cause I had no idea.* **P37, mother, telephone interview.**	**Parent questionnaire statement- I was initially surprised to find out that my child had already been entered into FiSh:** 13 (28.9%) agreed, 19 (42.2%) neither agreed nor disagreed, 2 (4.4%) disagreed.
	RWPC in FiSh was acceptable	(Discussed in 20 interviews)<br>*I think it was dealt with quite well. Obviously you need to do the research, and we weren't bothered there and then when he was being treated, which was quite good, and then they had the decency to come up and explain what has happened. So I didn't have a problem with that at all.* **P07,**	**Parent questionnaire statement- I understood why consent for my child's participation in FiSh was sought after the treatment had been given:** 44 (97.8 %) agreed, 1 (2.2 %) neither agreed nor

	**mother, telephone interview.**<br><br>*Well I am, and was, fine with it, because they had to do what they had to do and then afterwards…It was me that had to sign for them continue with the study, so I didn't see an issue with it.* **P15, mother, telephone interview.**	disagreed, 0 (0 %) disagreed.<br><br>**Parent questionnaire statement- I was satisfied with the deferred consent process in FiSh:** 39 (86.7%, agreed, 6 (13.3), neither agreed nor disagreed, 0 (0) disagreed.
Acceptable as fluid bolus resuscitation is not a drug or novel treatment	(Discussed in 5 interviews)<br>*Um, if it's a trial, I would want to know if it was medicine or drugs or if it was unknown surgery. So, unknown territory. The fluids, they're going to get fluids anyway so for me, [research without prior consent] was not even vaguely a concern. I think if they'd said we were going to trial a new medicine, I would want a bit more information on that.* **P40, mother, telephone interview.**<br><br>*It's just a different method. They're just the same item, as in two lots of 10, one lot of 20, which to my knowledge doesn't really make a massive difference.* **P33, mother, telephone interview.**	No quantitative data for this sub theme

**Clear explanation assisted understanding**	(Discussed in 13 interviews) *but I wasn't sort of concerned or anything, and then they explained everything really clearly.* **P12, mother, telephone interview.**<br><br>*Oh yes. The next day we got a full explanation of what the study was, why they were doing it, what they were hoping to find out, why I hadn't been told in the emergency room. It all made perfect sense to me.* **P13, mother, telephone interview.**	**Parent questionnaire statement- The information I received about FiSh was clear and straightforward to understand:** 42 (93.2%) agreed, 3 (6.7%) neither agreed nor disagreed, 0 (0%) disagreed.
**Child's survival and impact on parental acceptability**	(Discussed in 15 interviews) *I think that again it'd be down to outcome. If you have a positive outcome, I think the reaction would be completely different than if you had a negative outcome…it could cause an awful lot of ongoing pain, wondering, you know, whether or not that was a factor that tipped the balance.* **P06, mother, telephone interview.**<br><br>*No, I'm pretty sure that if, if they started to do the FiSh and then something went completely wrong, I'm sure rather than stick to the trial, they'd put the patient first and think, right we need to*	**Parent questionnaire. Reasons for consenting to the FiSh pilot trial. Option- My child recovered:** 22 (57.8 %) identified this a reason for consenting, 0 (0%) identified this as a main reason for consenting.

	do this, forget looking at the clock, this needs to be done. **P37, mother, telephone interview.**	
**Timing of FiSh discussion was appropriate**	(Discussed in 9 interviews)<br>*They made sure that it was the right time.* **P03, mother, telephone interview.**<br>*Yeah, it was, it was fine. It was definitely probably 24 hours after sort of the initial, um, scary period.* **P01, mother, telephone interview.**	**Parent questionnaire statement- The practitioner checked that it was a convenient time to discuss research before discussing FiSh**: 41 (91.1%) agreed, 2 (4.4 %) neither agreed nor disagreed, 2 (4.4 %) disagreed.<br><br>**Parent questionnaire statement- It was difficult to take in the information I was given about FiSh:** 1 (2.2%), agreed, 6 (13.3%), neither agreed nor disagreed, 38 (84.4) disagreed.
**Parental upset when hearing their child had sepsis during a FiSh trial discussion**	(Discussed in 4 interviews, 1 free text questionnaire response)<br>*I just heard sepsis and then I almost hit the floor.* **P39, mother, telephone interview.**<br><br>*What could have been improved was to make sure the parents know what actually happened in the situation before speaking about a trial.* **P26, mother, telephone interview.**	No quantitative data for this sub theme

		Example quotes from staff interviews and focus groups (n= 7 interviews and 3 focus groups)	*From voting keypad facilitated questions in staff focus groups (n=20)*
**Site training and resources**	Sufficiently prepared staff for the pilot trial	(Discussed in 3 focus groups, 7 interviews)<br>*It (training) made me feel confident enough to go and do the training for the extended team*. **P02, focus group 1.**<br><br>*you've got to have the background knowledge, so you've got to sort of attend the training to know what you're doing to fully understand why you're doing it, but also to have the reminder bits of the bits that are important to explain to the families at the point at which you're getting consent*. **P01, focus group 1.**	**Key pad question- Do you think the FiSh training prepared you for recruitment and consent in FiSh?** 20 (100%) yes, 0 (0%) no.
**Support for RWPC in FiSh**	Initial concerns resolved with experience	(Discussed in 3 focus groups, 7 interviews)<br>*And obviously the first deferred consent… you know it's the see one, do one, type thing and then I think you realise oh, actually it is okay*. **P02, focus group 1.**<br><br>*I was quite anxious about how they'd react, and I think the biggest thing that helped me was hearing that there'd been views from parents that they*	**Key pad question- Before FiSh, did you have any experience of research without prior consent?** 15 (75%), 5 (25%) no.<br><br>**Key pad question- Have your views on research without prior consent changed over time?**

		actually were happy about it…but both of them (parents of two recruited patients) were really, really understanding and recognised why we hadn't asked them at the time. **P18, focus group 3**.	14 (70%) yes, 6 (30%) no.
**Screening and randomisation process**	Missed patients due to lack of staff awareness	(Discussed in 2 focus groups, 4 interviews)<br>*It was a locum, it was a locum SHO on, with a brand new registrar, so there was nobody in paediatrics who was trained but it wasn't identified either by any ED staff.* **P24, telephone interview.**<br><br>*We have got a big poster down there but maybe we need to think...of where it is. It's on the back wall.* **P10, focus group 2.**	**Key pad question- Do you think the screening process could be improved?** 13 (65%) yes, 7 (35%) no.
	Randomisation process worked well	(Discussed in 2 focus groups, 3 interviews)<br>*The system is very much in place. I do like in ED the system where we use the sealed envelopes because it's quick and easy.* **P23, telephone interview.**<br><br>I think that the mechanism is very straightforward. You know, you pick up an envelope. **P21, telephone interview.**	**Key pad question- Do you think the randomisation process could be improved?** 6 (30%) yes, 14 (70%) no.
**Protocol adherence**	Lack of equipoise due to a belief that restrictive fluid bolus	(Discussed in 3 focus groups, 2 interviews)<br>*The child was allocated to 10 or 20, I think it was 10, but the doctor who was treating them decided I don't want them to*	**Key pad question- Have you experienced any difficulties adhering to the protocol?** 6 (30%)

	resuscitation is safer	*have 10, I want them to have five.* **P6, focus group 1.**<br><br>*The registrar gave 20 ml per kilo and then gave a further 10, rather than giving 20 and 20. I think that's, you know, so what we've got here is examples of clinical creep of lower volume fluid resuscitation in the absence of evidence.* **P22, telephone interview.**	yes, 5 (25%) no, 9 (45%) missing.
	Administering 20ml/kg boluses within the 15-minute cycles	(Discussed in 2 focus groups, 2 interviews)<br>*Giving the bolus within 15 minutes of randomisation, I just don't think that's feasible. I don't think I've looked at the CRF for a single child who has actually been achieved within the fifteen minute window.* **P19, focus group 3.**<br><br>*The calibre of the cannula allows it to be possible but you can't necessarily push it that quickly in through a cannula of that size in every child, depending on how it's placed and things like that*. **P18, focus group 3.**	
**Problems completing the Case Report Form**		(Discussed in 3 focus groups, 5 interviews)<br>*I'm not expecting people to stop resuscitating to start filling in paperwork, but, you know, just sort of a better awareness maybe of what we need might improve what people are actually recording when they're*	**Key pad question- Is the Case Report Form easy to use?** 6 (30%) yes, 8 (40%) no, 6 (30%) missing

recording things. **P24, telephone interview.**

It looks quite daunting, so maybe if there was a way of trimming it down so that only the bare essentials that are needed from the emergency. **P25, telephone interview.**

FiSh, Fluids in Shock; RWPC, Research without prior consent. Brackets are used around explanatory text in quotes.
